# Supplementary material for: Possible Involvement of the Upregulation of ΔNp63 Expression Mediated by HER2-Activated Aryl Hydrocarbon Receptor in Mammosphere Maintenance
Source: Int J Mol Sci. 2022 Oct 11;23(20):12095. doi: 10.3390/ijms232012095 (PMC9603582; doi:10.3390/ijms232012095)
Supplement: Supplementary file 1 [file ijms-23-12095-s001.zip › ijms-1870410-supplementary.pdf]

Supplementary figure S1

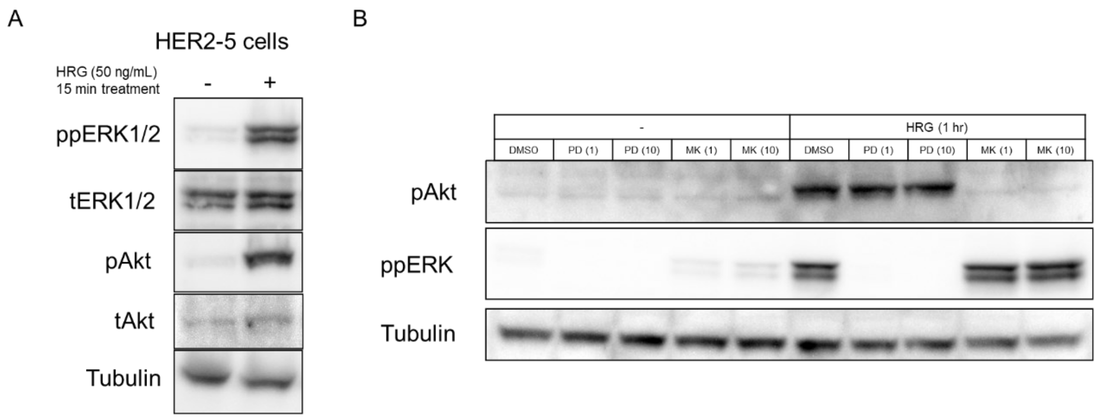

Supplementary figure S1 (A) HER2-5 cells were cultured in a medium with low serum (2% csFBS DMEM) for 24 h and treated with HRG (50 ng/mL) for 15 min. Subsequently, the cells were harvested, whole-cell lysates (10 μg) were resolved by sodium dodecyl sulfate-polyacrylamide gel electrophoresis, and proteins were detected by immunoblot analysis using antibodies against Phospho-p44/42 MAPK (ppERK1/2), total ERK1/2 (tERK1/2), Phospho-Akt (pAkt), total Akt (tAkt) and α-tubulin. (B) HER2-5 cells were cultured in a medium with low serum (2% csFBS DMEM) for 24 h and pre-treated with PD0325901 (1 μM, 10 μM), MK-2206 (1 μM, 10 μM) or DMSO (solvent control) for 1 h. And then, the cells were stimulated with HRG (50 ng/mL) or vehicle (water) for 1 h. Subsequently, the cells were harvested, whole-cell lysates (10 μg) were resolved by sodium dodecyl sulfate-polyacrylamide gel electrophoresis, and proteins were detected by immunoblot analysis using antibodies against ppERK1/2, pAkt and α-tubulin.

Supplementary figure S2

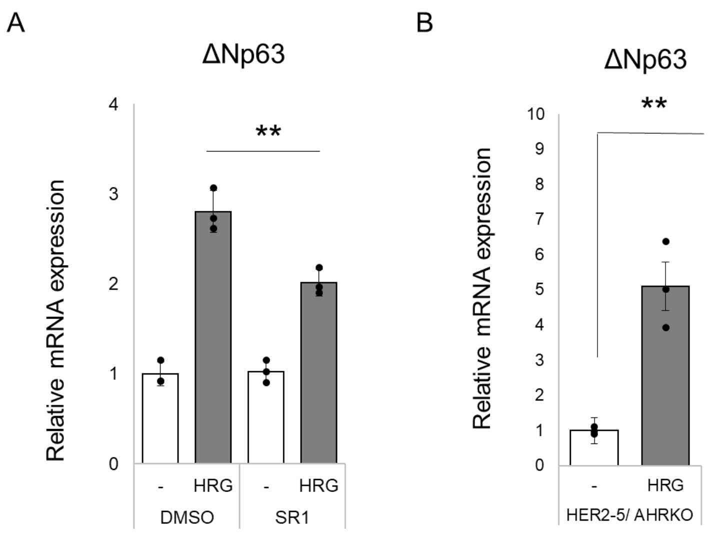

Supplementary figure S2 (A) HER2-5 cells were cultured in a medium with low serum (2% csFBS DMEM) for 24 h and pre-treated with SR1 (1 μM) or DMSO (solvent control) for 1 h. And then, the cells were stimulated with HRG (50 ng/mL) or vehicle (water) for 6 h, and relative mRNA levels were determined. (mean ± S.D., n = 3). **, $p < 0.01$ (Student's t-test). The results are representative of two independent biological experiments. (B) HER2-5/AHRKO cells were cultured in a medium with low serum (2% csFBS DMEM) for 24 h and treated with HRG (50 ng/mL) for 3 h. Subsequently, the cells were harvested, and ΔNp63 mRNA level was determined by RT-qPCR analysis and normalized to that of B2M mRNA levels. Values are expressed as relative values (those in HER2-5 cells were set at 1) and mean ± S.D. (n = 3) **, $p < 0.01$ (Student's t-test).
